# Machine learning in dementia risk identification using routinely collected data

**DOI:** 10.1002/alz70856_105348

**Published:** 2026-01-07

**Authors:** Cristian Gonzalez Prieto, Gillian Dobbie, Claudia Rivera Rodriguez, Daniel Wilson, Susan Yates, Sarah J Cullum

**Affiliations:** ^1^ The University of Auckland, Auckland, Auckland, New Zealand

## Abstract

**Background:**

Dementia is a major global public health challenge, affecting approximately 55 million people worldwide, with an estimated 10 million new cases annually (WHO). In New Zealand, prevalence estimates based on national datasets suggest that dementia affects 3.8%–4.0% of individuals aged 60 and older. When accounting for undiagnosed cases using a capture‐recapture method, this estimate increases to 9.2% (95% CI: 8.9%–9.6%), with disproportionately higher rates among Māori and Pacific populations. However, nearly 50% of dementia cases remain undiagnosed, limiting timely interventions and increasing healthcare costs. There is an urgent need for scalable, data‐driven solutions to improve dementia identification. This study aimed to develop accurate machine learning models for dementia identification using routinely collected health data.

**Method:**

Routinely collected health data from the Te Whatu Ora Counties Manukau population (aged 65+) were analysed, incorporating sociodemographic and clinical variables (both longitudinal and cross‐sectional). Dementia status was determined based on pharmacy records (antidementia drug prescriptions), interRAI assessments (dementia‐related evaluations), and hospitalization records (ICD‐10 codes related to dementia). A nested one‐to‐one case‐control design was implemented, retaining pre‐diagnostic information across six‐time windows before diagnosis (0 days, 6 months, 1 year, 3 years, 5 years, and 8 years). Deep learning models were trained using a training/validation/testing framework.

**Result:**

The models achieved an accuracy of 80.47% (95% CI: 80.23–80.72) when using data immediately before diagnosis with a sensitivity of 71.77% (95% CI: 71.73–71.81) and specificity of 87.27% (95% CI: 87.24–87.30). The highest performance was achieved when all available features were included.

Key predictive features included Aged Residential Care (ARC)‐related factors, comorbidities (ICD‐10 codes), and ethnicity. Among pharmacy‐related variables, analgesics, diuretics, antithrombotics, anti‐epileptic drugs, and diabetes treatments were highly relevant. The number of ophthalmology and cardiology appointments, along with delirium‐related features, also contributed significantly.

**Conclusion:**

Machine learning applied to routinely collected health data offers a powerful, scalable approach to dementia case‐finding, improving early identification and facilitating timely interventions. These models have the potential to enhance clinical decision‐making, optimize healthcare resources, and ultimately improve outcomes for individuals living with undiagnosed dementia.

